# ﻿Description of three new species of Spartaeini (Araneae, Salticidae, Spartaeinae) from China and Malaysia

**DOI:** 10.3897/zookeys.1232.146855

**Published:** 2025-03-20

**Authors:** Yi Ni, Kun Yu, Junxia Zhang

**Affiliations:** 1 Key Laboratory of Zoological Systematics and Application, College of Life Sciences, Hebei University, Baoding, Hebei 071002, China Hebei University Baoding China; 2 Hebei Basic Science Center for Biotic Interaction, Hebei University, Baoding, Hebei 071002, China Hebei University Baoding China

**Keywords:** Basal lineages, jumping spider, morphology, taxonomy

## Abstract

Three new species of Spartaeini from China and Malaysia are described: *Calxattusdengba***sp. nov.** (♂), *Spartaeussiloi***sp. nov.** (♂♀), and *Taraxellachrisfehni***sp. nov.** (♂♀). *Calxattusdengba* is the second discovered species of the genus in the world. The genus *Taraxella* is illustrated with a colour plate for the first time.

## ﻿Introduction

The family Salticidae is renowned for its remarkable species diversity, largely attributed to the subfamily Salticinae, which comprises over 6000 of the nearly 7000 known salticid species (WSC 2025). In contrast, the remaining six salticid subfamilies, often referred to as the “basal lineages”, consist of only a few hundred species across approximately 50 genera (Maddison 2015; WSC 2025). Among these, the tribe Spartaeini of the subfamily Spartaeinae is the most diverse, yet it only includes 134 species in 19 genera, all restricted to the Old World (WSC 2025). Notably, 11 of these genera have not had any new species discovered in the last decade (WSC 2025), likely due to insufficient exploration of those areas. The latest species described for the genera *Taraxella* Wanless, 1984 and *Holcolaetis* Simon, 1886 date back to the 1980s (Wanless 1985, 1987). Furthermore, most genera within this tribe exhibit relatively low species diversity, with about one-third being oligotypic or monotypic. Examples include *Wanlessia* Wijesinghe, 1992 (2 spp.), *Veissella* Wanless, 1984 (2 spp.), *Sparbambus* Zhang, Woon & Li, 2006 (2 spp.), *Sonoita* Peckham & Peckham, 1903 (2 spp.), *Paracyrba* Żabka & Kovac, 1996 (1 sp.), and *Calxattus* Wang, Yu & Zhang, 2023 (1 sp.) (WSC 2025).

Here, we describe three new species of Spartaeini: *Calxattusdengba* sp. nov. (male from Xizang, China), *Spartaeussiloi* sp. nov. (both sexes from Guangxi, China), and *Taraxellachrisfehni* sp. nov. (both sexes from Peninsular Malaysia). Notably, *C.dengba* marks the second species of *Calxattus* to be described. Additionally, it has been 38 years since a new species of *Taraxella* was last documented, and this study provides the first colour figure plate for the genus. This study aims to advance our knowledge of the species diversity of Spartaeini.

## ﻿Material and methods

Specimens preserved in 75% or 95% ethanol were examined under a Nikon SMZ 1500 stereomicroscope and measured using a Leica M205A stereomicroscope. All measurements were taken in millimeters with the dedicated measurement tool in Leica LAS v. 4.3 software. Female genitalia were cleared in a pancreatin solution (Álvarez-Padilla and Hormiga 2007). The setae of male palp were mostly removed using a Chinese acupuncture needle to ensure that its complex palpal structures were not obscured. Photographs of the ethanol-immersed bodies were captured using a Leica M205A stereomicroscope equipped with a Leica DFC550 CCD camera and stacked using LAS v. 4.3 software. Images of the genitalia and other details were captured using an Olympus BX53 microscope equipped with a Kuy Nice CCD camera. The resulting image stacks were then processed using Helicon Focus v. 8 software. Final images were retouched in Adobe Photoshop CC ©2023. All specimens examined are deposited in the Museum of Hebei University (MHBU; Baoding, China).

Terminology in this study mainly followed Wanless (1984, 1987) and Wang et al. (2023), with a few exceptions (see comments for *Spartaeussiloi* sp. nov. and *Taraxellachrisfehni* sp. nov.). Abbreviations used in this study: **AG**, accessory gland; **ALE**, anterior lateral eye; **AME**, anterior median eye; **C**, functional conductor; **CD**, copulatory duct; **CO**, copulatory opening; **Cy**, cymbium; **DH**, distal haematodocha; **dITA**, dorsal intermediate tibial apophysis; **E**, embolus; **EG**, embolic guide; **FD**, fertilization duct; **ITA**, intermediate tibial apophysis; **M**, membrane of distal haematodocha; **MA**, median apophysis; **MTA**, membranous tegular apophysis; **PLE**, posterior lateral eye; **PME**, posterior median eye; **RTA**, retrolateral tibial apophysis; **SD**, sperm duct; **St**, subtegulum; **T**, tegulum; **TA**, tegular apophysis; **TD**, tegular depression; **vITA**, ventral intermediate tibial apophysis; **VTA**, ventral tibial apophysis; **X**, tegular apophysis ‘X’; **Y**, tegular apophysis ‘Y’.

## ﻿Taxonomy

### ﻿ Calxattus

Taxon classificationAnimaliaAraneaeSalticidae

Wang, Yu & Zhang, 2023

3A5A3DC6-881F-5D2C-BA1A-C13D362A4702

Calxattus Wang, Yu & Zhang, 2023: 9.

#### Type species.

*Spartaeusserratus* Yang, Liu, Liu & Peng, 2017, by original designation.

### ﻿ Calxattus dengba sp. nov.

Taxon classificationAnimaliaAraneaeSalticidae

123D8386-5A46-52BD-A036-76D0A1F27C00

https://zoobank.org/655EF590-7FF4-4ED5-AFEF-52392536DE9F

**Figure 1.**
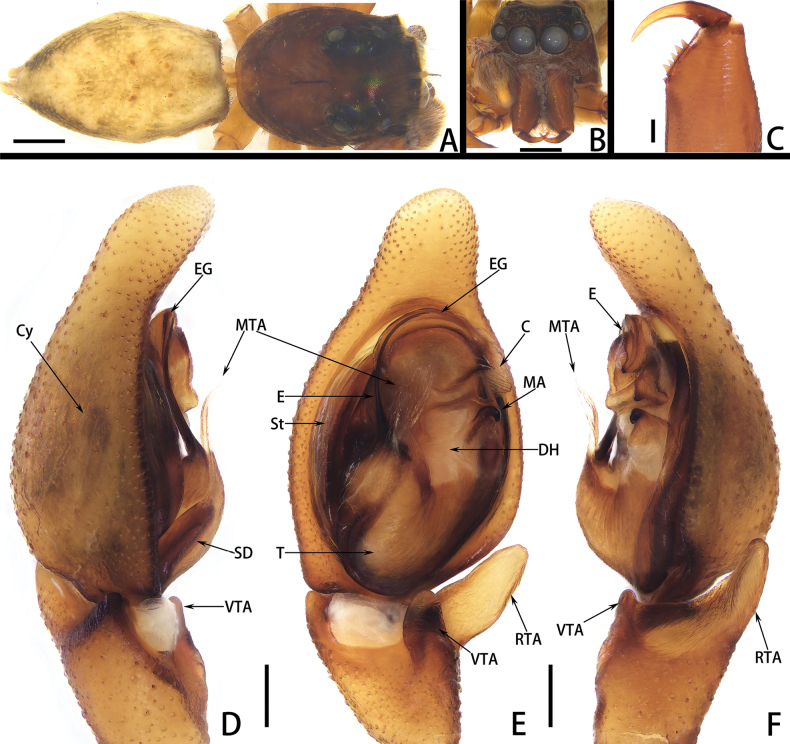
*Calxattusdengba* sp. nov., habitus of holotype male (**A, B**), cheliceral teeth (**C**), palp of holotype male (**D–F**); in dorsal (**A**), front (**B**), prolateral (**D**), ventral (**E**), retrolateral (**F**) and back (**C**) view. Scale bars: 1 mm (**A, B**); 0.2 mm (**C–F**).

**Figure 2.**
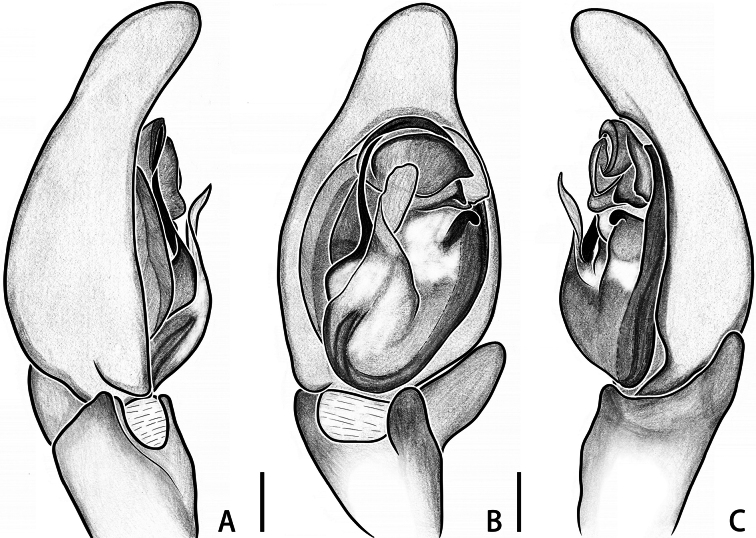
*Calxattusdengba* sp. nov., palp of holotype male (**A–C**); in prolateral (**A**), ventral (**B**) and retrolateral (**C**) view. Scale bars: 0.2 mm (**A–C**).

Figs 1 , 2 Common name. 僜巴岩跳蛛

#### Type material.

***Holotype***: China • ♂ (MHBU-ARA-00027415); Xizang Autonomous Region: Nyingchi City, Zayu County, Xia Zayu Town; 9.VIII.2002; M. Zhu leg.

#### Etymology.

The specific epithet is derived from the Dengba people, who live in Zayu County, the type locality of the new species. Noun in apposition.

#### Diagnosis.

The male of the new species can be distinguished from its sole congeneric species, *C.serratus*, by the following: (1) the RTA lacks processes on its prolateral and retrolateral edges (Figs 1E, 2B; vs. in *C.serratus*, the RTA carries median processes on both prolateral and retrolateral edges; see Wang et al. 2023: figs 5B, 7B); (2) the embolus is relatively short, originates from the median portion of the palpal bulb and terminates near the midpoint of the lower edge of the groove-shaped embolic guide (Figs 1E, 2B; vs. in *C.serratus*, the embolus is long, originates from the lower portion of the bulb and terminates through the groove-shaped embolic guide near the functional conductor; see Wang et al. 2023: figs 5B, 7B); and (3) the median apophysis is located in the upper half of the retrolateral part of the bulb (Figs 1E, 2B; vs. in *C.serratus*, the median apophysis is located in the lower half of the retrolateral part of the bulb; see Wang et al. 2023: figs 5B, 7B).

#### Description.

**Male.** Measurements of holotype: total length 7.26. Carapace 3.52 long, 2.47 wide. Abdomen 3.34 long, 2.28 wide. Eye measurements: AME 0.63, ALE 0.26, PME 0.43, PLE 0.37. Leg measurements: I 14.18 (3.78, 1.35, 4.47, 2.91, 1.67), II 7.89 (2.80, 0.96, 2.28, 1.35, 0.50), III 7.74 (2.41, 0.87, 1.86, 1.84, 0.76), IV 11.39 (3.07, 0.83, 3.04, 3.27, 1.18); leg formula 1423. Chelicera (Fig. 1C) brown, with six promarginal and seven retromarginal teeth. Carapace (Fig. 1A, B) reddish-brown, eye surroundings black, clypeus and eye surroundings covered by sparse white setae, fovea dark and longitudinal slit-like. Endites and labium yellowish-brown, with brown setae at distal end; sternum flat and creamy yellow, covered with white and brown setae. Legs (Fig. 1A) dark yellow, bearing long and thin spines; leg I extra long. Abdomen (Fig. 1A) pale yellow and elongated oval, surface faded; dorsal side with visible grey bands at edge, ending near spinnerets; short yellow setae covered on dorsal lateral sides of anterior half; ventral side with fine or flattened setae in middle part. Spinnerets (Fig. 1A) yellow, covered with white setae.

Palp (Figs 1D–F, 2A–C): embolus thread-like, terminating near midpoint of embolic guide in ventral view; median apophysis black, hook-like in ventral view, located in upper half of retrolateral part of bulb, near functional conductor; functional conductor fan-shaped, with plow-like furrows on surface; membranous tegular apophysis transparent, curved in middle portion, originating from tegular apex and covering half of basal embolus; retrolateral and ventral tibial apophyses finger-like in ventral view, surface of retrolateral tibial apophysis with fingerprint-like patterns.

**Female.** Unknown.

#### Distribution.

China (Xizang).

### ﻿ Spartaeus

Taxon classificationAnimaliaAraneaeSalticidae

Thorell, 1891

B3AFF89D-F73B-53C8-8E88-492087A14C62

Spartaeus Thorell, 1891: 137.

#### Type species.

*Boethusspinimanus* Thorell, 1878, by original designation.

### ﻿ Spartaeus siloi sp. nov.

Taxon classificationAnimaliaAraneaeSalticidae

106A82DC-C8FE-5F1B-BCD2-EE4A99B8F0F0

https://zoobank.org/531DF00D-0A70-4AB3-B507-89B1EEC6A5AE

**Figure 3.**
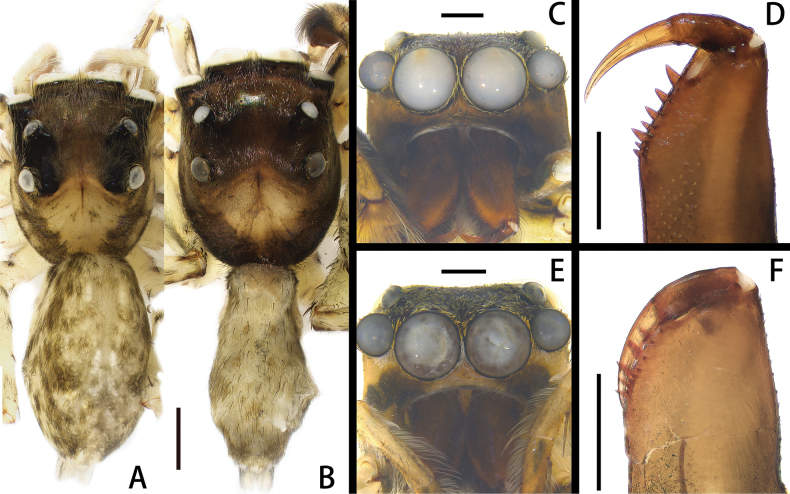
*Spartaeussiloi* sp. nov., habitus of holotype male (**B, C)** and paratype female (**A, E**), cheliceral teeth of holotype male (**D**) and paratype female (**F**); in dorsal (**A, B**), front (**C, E**) and back (**D, F**) view. Scale bars: 0.1 mm (**A, B**); 0.5 mm (**C–F**).

**Figure 4.**
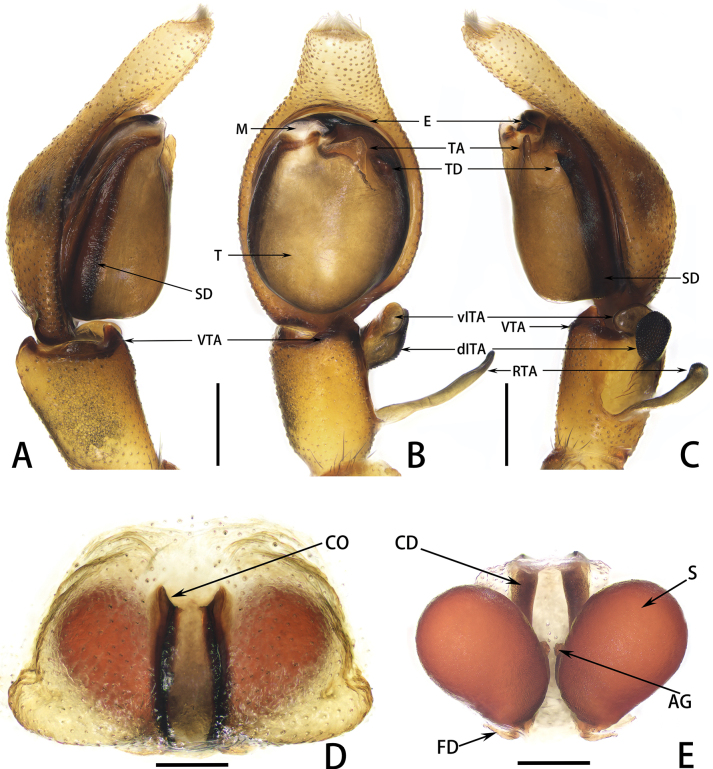
*Spartaeussiloi* sp. nov., palp of holotype male (**A–C**), epigyne (**D**) and vulva (**E**) of paratype female; in prolateral (**A**), ventral (**B, D**), retrolateral (**C**) and dorsal (**E**) view. Scale bars: 0.2 mm (**A–E**).

**Figure 5.**
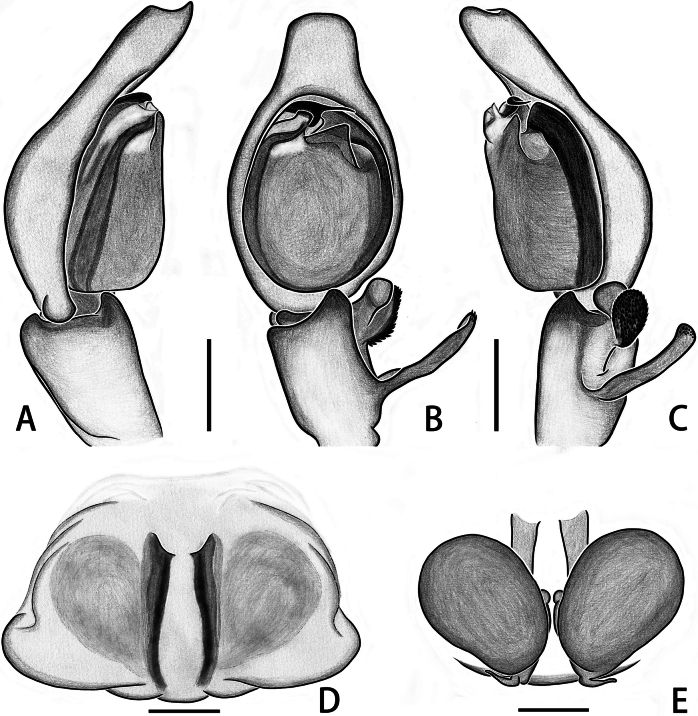
*Spartaeussiloi* sp. nov., palp of holotype male (**A–C**), epigyne (**D**) and vulva (**E**) of paratype female; in prolateral (**A**), ventral (**B, D**), retrolateral (**C**) and dorsal (**E**) view. Scale bars: 0.2 mm (**A–E**).

Figs 3 , 4 , 5 Common name. 诗雷散蛛

#### Type material.

***Holotype***: China • ♂ (MHBU-ARA-00027416); Guangxi Zhuang Autonomous Region, Chongzuo City, Daxin County, Longdian; 22.6931°N, 107.036975°E, 211 m elev.; 27.IV. 2024; W. Wang leg. ***Paratypes***: China • 1♀ (MHBU-ARA-00027417); same data as holotype; 1♂ (MHBU-ARA-00027538); Guangxi Zhuang Autonomous Region Autonomous Region, Chongzuo City, Daxin County, Xinkang; 22.6538°N, 107.0755°E, 145 m elev.; 12.V.2024; J. Zhang, K. Yu, Z. Yang, Y. Ni & Y. Li leg.

#### Etymology.

The specific epithet is derived from “Si Loi”, which is a unique artistic style of Zhuang folk songs in Daxin County (the type locality). Noun in apposition.

#### Diagnosis.

The new species resembles *S.platnicki* Song, Chen & Gong, 1991 in the shape of the palpal bulb and copulatory duct, but it can be distinguished by the following: (1) the length of the embolus is much less than half of the bulb width (Figs 4B, 5B; vs. in *S.platnicki*, the length of the embolus is more than half of bulb width; see Lin et al. 2023: fig. 18A); (2) the end of the retrolateral tibial apophysis is relatively straight (Figs 4B, 5B; vs. in *S.platnicki*, the end of the retrolateral tibial apophysis is strongly curved; see Lin et al. 2023: fig. 18A); (3) the ventral intermediate tibial apophysis is rounded at the distal end in ventral view (Figs 4B, 5B; vs. in *S.platnicki*, the ventral intermediate tibial apophysis is sharp at the distal end in ventral view; see Lin et al. 2023: fig. 18A); and (4) the two copulatory ducts are nearly parallel (Figs 4D, 5D; vs. curved posteriorly in *S.platnicki*; see Lin et al. 2023: fig. 19A).

#### Description.

**Male.** Measurements of holotype: total length 7.19. Carapace 3.42 long, 2.37 wide. Abdomen 3.58 long, 1.56 wide. Eye measurements: AME 0.81, ALE 0.45, PME 0.30, PLE 0.30. Leg measurements: I 15.55 (4.43, 1.28, 4.70, 3.7, 1.44), II 9.29 (2.30, 0.91, 2.61, 2.40, 1.07), III 10.05 (2.67, 0.89, 2.38, 2.53, 1.58), IV 13 (3.16, 1.07, 3.39, 4.19, 1.19); leg formula 1432. Chelicera (Fig. 3D) dark brown, with seven promarginal and eight retromarginal teeth. Carapace (Fig. 3B, C) yellow, margin and eye surroundings blackish-brown, anterior lateral margin deep black, eye field with black setae, eye surroundings covered by yellow and white setae, fovea dark and slit-like; endites and labium yellowish-brown, with black setae at distal end, endites slender. Legs (Fig. 3B) with long and thin spines, coxa and trochanter off-white, femur to tibia with obvious grey stripes on anterior and posterior sides. Abdomen (Fig. 3B) yellow, black strips interspersed with white setae around margins, broken near spinnerets; dorsal side with tawny patches and black setae; ventral side off-white, with white setae on both sides, center covered with greyish-white scales. Spinnerets (Fig. 3B) white, spinnerets lateral inward white, lateral exterior with dark brown patches, covered with white setae.

Palp (Figs 4A–C, 5A–C): embolus thin and short, sheep-horn-like in retrolateral view, base of embolus hidden behind membrane formed by distal haematodocha; tegular oval, surface smooth; tegular apophysis triangular, weakly sclerotized in ventral view; ventral tibial apophysis thick, triangular in ventral view; retrolateral tibial apophysis soft and movable, long and flat in ventral view, with small processes on distal end; dorsal intermediate tibial apophysis blackish-brown, with snake-scale-like processes on outer surface, edge wide and flat; ventral intermediate tibial apophysis yellowish-brown, rounded at distal end in ventral view; distal end of cymbium with grey hair tuft.

**Female.** Measurements of paratype: total length 6.55. Carapace 2.92 long, 2.31 wide. Abdomen 3.33 long, 2.07 wide. Eye measurements: AME 0.68, ALE 0.40, PME 0.28, PLE 0.39. Leg measurements: I 9.15 (2.72, 1.03, 2.70, 1.84, 0.86), II 7.16 (2.25, 0.76, 1.88, 1.53, 0.74), III 7.45 (2.19, 074, 1.84, 1.83, 0.85), IV 8.68 (2.85, 0.72, 2.67, 1.52, 0.92); leg formula 1432. Chelicera (Fig. 3F) brown, with six promarginal and eight retromarginal teeth. Habitus (Fig. 3A, E) similar to that of male, but different in: carapace paler at margin forming a black band from anterior lateral eyes to rear edge of carapace; with a black band in lower position between the anterior median eyes and the posterior lateral eyes; black strips on lateral dorsal side of abdomen not continuous, but with intermittent small black bands.

Epigyne (Figs 4D, E, 5D, E): epigynal plate oval, slightly raised in a mound-like shape; copulatory openings small, slit-shaped in ventral view, anteriorly located; copulatory ducts extending to posterior part, dark and slender; spermathecae reddish-brown, large and kidney-shaped; fertilization ducts short, connecting with spermathecae at posterior part; accessory glands located centrally at inner margins of spermathecae, partly hidden by spermathecae in dorsal view.

#### Distribution.

China (Guangxi).

#### Comments.

Following a previous study on *Spartaeus* (Yang et al. 2017: figs 4, 8, 10), we labelled the weekly sclerotized apophysis derived from the tegulum of male palpal bulb as TA. This structure was identified as M_3_ in Wanless (1984). Additionally, the membranous patch of the distal haematodocha at the base of embolus is referred to here as M, aligning with the designation of M_1_ in Wanless’s (1984) study on *Spartaeus*. In the redescription of *S.platnicki* Song, Chen & Gong, 1991, the marked TA (Lin et al. 2023: fig. 18) is likely mislabelled and probably should be M in this study, considering its position at the base of embolus.

### ﻿ Taraxella

Taxon classificationAnimaliaAraneaeSalticidae

Wanless, 1984

C84C49CE-95B6-553B-95A7-0227059873C0

Taraxella Wanless, 1984: 155.

#### Type species.

*Taraxellasolitaria* Wanless, 1984, by original designation.

### ﻿ Taraxella chrisfehni sp. nov.

Taxon classificationAnimaliaAraneaeSalticidae

D0267696-D6DF-5CC5-8528-2E29BD8C475C

https://zoobank.org/C96EB08B-A1FB-4E1C-A05F-787B5147C599

**Figure 6.**
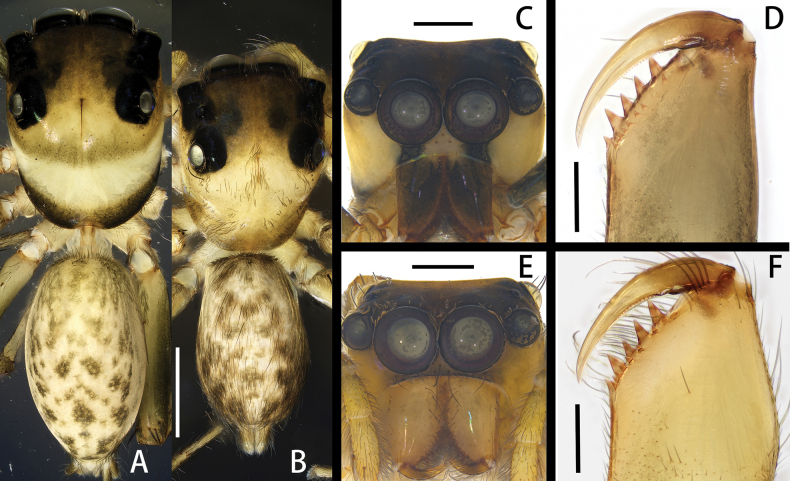
*Taraxellachrisfehni* sp. nov., habitus of holotype male (**A, C**) and paratype female (**B**, **E**), cheliceral teeth of holotype male (**D**) and paratype female (**F**); in dorsal (**A, B**), front (**C, E**) and back (**D, F**) view. Scale bars: 1 mm (**A, B**); 0.5 mm (**C, E**); 0.2 mm (**D, F**).

**Figure 7.**
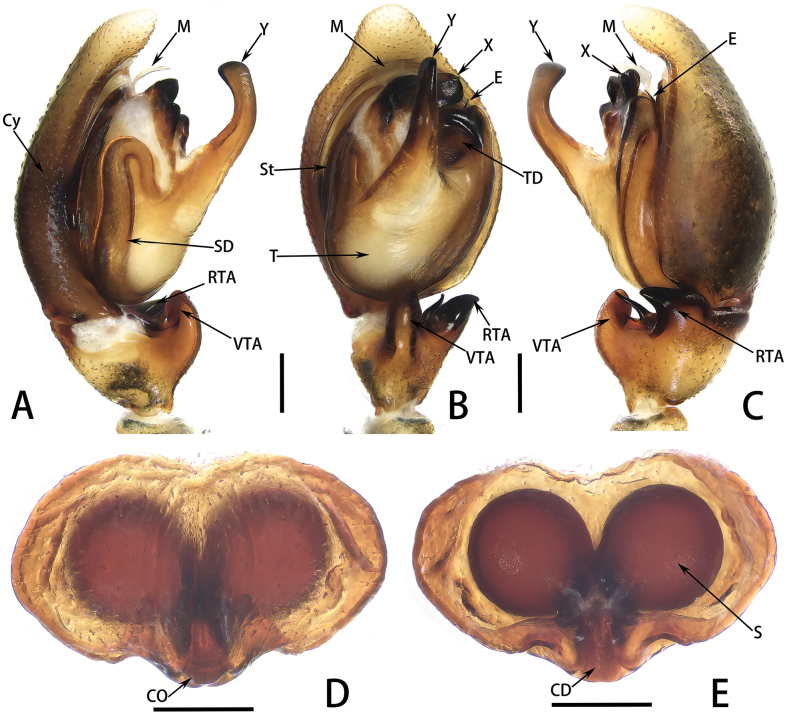
*Taraxellachrisfehni* sp. nov., palp of holotype male (**A–C**), epigyne (**D**) and vulva (**E**) of paratype female; in prolateral (**A**), ventral (**B, D**), retrolateral (**C**) and dorsal (**E**) view. Scale bars: 0.2 mm (**A–E**).

**Figure 8.**
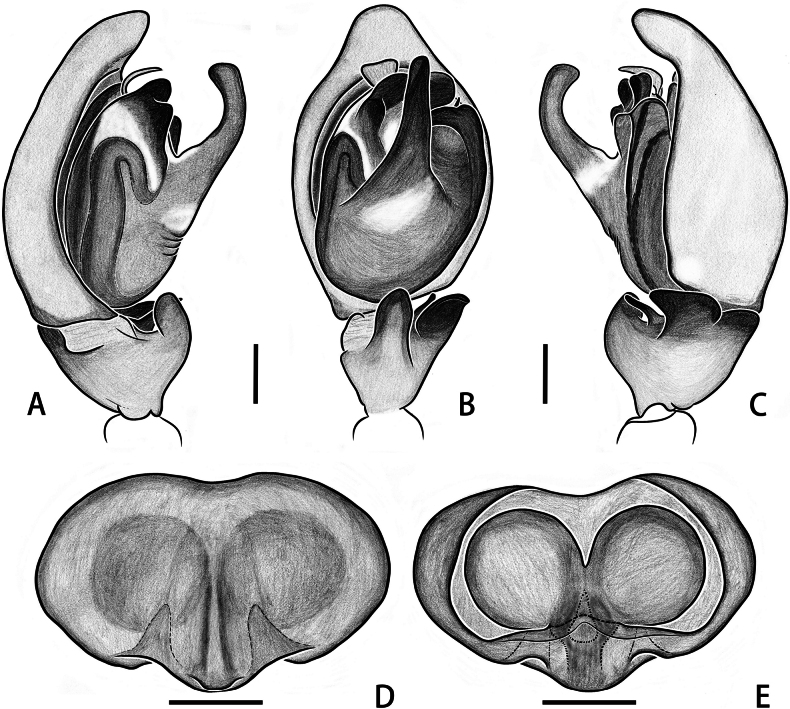
*Taraxellachrisfehni* sp. nov., palp of holotype male (**A–C**), epigyne (**D**) and vulva (**E**) of paratype female; in prolateral (**A**), ventral (**B, D**), retrolateral (**C**) and dorsal (**E**) view. Scale bars: 0.2 mm (**A–E**).

Figs 6 , 7 , 8 Common name. 克氏塔蛛

#### Type material.

***Holotype***: Malaysia • ♂ (MHBU-ARA-00021518); Kelantan, Gua Musang; 4.7635°N, 102.0055°E, 199 m elev.; 26.X.2015; Z. Gao, G. Huang & L. Wang leg. ***Paratype***: Malaysia • 1♀ (MHBU-ARA-00027418); same data as the holotype.

#### Etymology.

The specific epithet is derived from Chris Fehn, the percussionist and backing vocalist for the heavy metal band Slipknot from 1998 to 2019; the Pinocchio-style mask that Chris Fehn usually wears during his performances resembles the tegular apophysis ‘Y’ on the palpal bulb of the new species.

#### Diagnosis.

The new species resembles *T.solitaria* in the pattern of tegular apophyses ‘X’ and ‘Y’, but it can be distinguished from *T.solitaria* by the following: (1) the ‘Y’ apophysis is slender, originates from the center of the palpal bulb, protrudes at a 45° angle toward the ventral distal end, bent in proximal 1/3 position from tip (Figs 7A–C, 8A–C; vs. in *T.solitaria*, the apophysis ‘Y’ is short and stout, originates from the prolateral part of the palpal bulb; see Wanless 1984: fig. 7G, F); (2) the sharp prong is short and slightly curved, positioned between the retrolateral tibial apophysis and the ventral tibial apophysis (Figs 7B, C, 8B, C; vs. in *T.solitaria*, the sharp prong is long and curved in an “S” shape, located dorsally to the retrolateral tibial apophysis; see Wanless 1984: fig. 7G, F); and (3) the membrane of distal haematodocha forms a fan-shaped structure that resembles an inverted trapezoid in ventral view (Figs 7B, 8B; vs. in *T.solitaria*, the fan-shaped structure resembles a rhomboid in ventral view; see Wanless 1984: fig. 7G)

#### Description.

**Male.** Measurements of holotype: total length 4.75. Carapace 2.24 long, 1.68 wide. Abdomen 2.16 long, 1.28 wide. Eye measurements: AME 0.55, ALE 0.28, PME 0.09, PLE 0.28. Leg measurements: I 5.28 (1.50, 0.50, 1.48, 1.22, 0.58), II 4.12 (1.18, 0.48, 1.17, 0.93, 0.36), III 4.49 (1.21, 0.45, 1.10, 1.25, 0.49), IV 4.44 (1.35, 0.41, 1.10, 1.05, 0.53); leg formula 1342. Chelicera (Fig. 6D) brown, bearing large black patch in anterior region, with five promarginal and nine retromarginal teeth. Carapace (Fig. 6A, C) with dark margins, and an off-white encircling band, eye field light brown; eye surroundings dark, with black strips beneath anterior median eyes connecting to carapace margin resembling tear stains; fovea brown and longitudinal slit-like; endites light brown, with black setae at distal end; labium pale yellow, with a black spot on surface. Legs (Fig. 6A) bearing long and thin spines, coxa and trochanter off-white, femur and tibia olive-brown. Abdomen (Fig. 6A) off-white on dorsal side, with mottled long black setae; clusters of dark brown setae adorning lateral sides; herringbone patterned lines arranged longitudinally, gradually decreasing in size from front to back in middle; ventral side off-white. Spinnerets (Fig. 6A) pale yellow, covered with black setae.

Palp (Figs 7A–C, 8A–C): embolus partly hidden by tegular apophysis ‘X’ and tegulum in ventral view; tegular apophysis ‘X’ dark, located at distal end of bulb, extending to retrolateral direction, forming three hill-like protrusions on surface in retrolateral view; tegular apophysis ‘Y’ long, bent in proximal 1/3 position from tip; membrane of distal haematodocha forming a transparent fan-like structure, bent ventrally; ventral tibial apophysis thick, bent dorsally, finger-like in ventral view, edge dark; retrolateral tibial apophysis dark, with a ventral depression, sheep-ear-like in ventral view; prong between VTA and RTA sharp, petiole-like.

**Female.** Measurements of paratype: total length 4.07. Carapace 1.90 long, 1.53 wide. Abdomen 2.09 long, 1.20 wide. Eye measurements: AME 0.50, ALE 0.06, PME 0.29, PLE 0.27. Leg measurements: I 3.76 (1.38, 0.46, 0.99, 0.66, 0.27), II 3.38 (1.17, 0.46, 0.90, 0.62, 0.23), III 4.03 (1.20, 0.39, 0.98, 0.87, 0.59), IV 5.49 (1.54, 0.48, 1.52, 1.49, 0.46); leg formula 4312. Chelicera pale yellow, with five promarginal and eight retromarginal teeth (Fig. 6F). Habitus (Fig. 6B, E) similar to that of male, but different in: carapace paler at margin, lacking obvious off-white band, clypeus narrow, faint black, lacking obvious black strips beneath anterior median eyes; femur and tibia consistent in colour with other leg segments.

Epigyne (Figs 7D, E, 8D, E): epigynal plate heart-shaped, equipped with a longitudinal furrow at posterior-middle part; two triangular concave cavities (possible epigynal pockets) at posterior end facing rearward; copulatory openings slit-shaped, located behind longitudinal furrow; copulatory ducts hidden beneath sclerotized integument and inconspicuous at dorsal view; spermathecae large and spherical, with middle parts closely adhering to each other; fertilization ducts and accessory glands not visible.

#### Distribution.

Malaysia (Kelantan).

#### Comments.

*Taraxella* has the peculiar tegular apophyses ‘X’ and ‘Y’, as well as the nearly concealed embolus (Wanless 1987). Tegular apophysis ‘Y’ is likely homologous to M_3_, which may also be denoted as TA (Wanless 1984). Nevertheless, the structure homologous to ‘X’ remains unclear, and the homology of different apophyses on male palpal bulb in Spartaeinae remains to be investigated. In this study, we followed the terminology in Wanless (1984) and employed ‘X’ and ‘Y’ to represent the two massive apophyses of the male palp.

## Supplementary Material

XML Treatment for Calxattus

XML Treatment for Calxattus dengba

XML Treatment for Spartaeus

XML Treatment for Spartaeus siloi

XML Treatment for Taraxella

XML Treatment for Taraxella chrisfehni

## ﻿Additional information

### Author ORCIDs

Yi Ni https://orcid.org/0009-0003-3239-3697

Kun Yu https://orcid.org/0000-0002-5517-4484

Junxia Zhang https://orcid.org/0000-0003-2179-3954

### Data availability

All of the data that support the findings of this study are available in the main text.
